# Management after initial surgery of nonfunctioning pituitary adenoma: surveillance, radiotherapy or surgery?

**DOI:** 10.1186/s13014-022-02133-z

**Published:** 2022-10-13

**Authors:** Thomas Charleux, Véronique Vendrely, Aymeri Huchet, Renaud Trouette, Amandine Ferrière, Antoine Tabarin, Vincent Jecko, Hugues Loiseau, Charles Dupin

**Affiliations:** 1 Radiotherapy Department, CHU Bordeaux, 33000 Bordeaux, France; 2 BRIC (BoRdeaux Institute of onCology), UMR1312, INSERM, University of Bordeaux, 33000 Bordeaux, France; 3 Endocrinology Department, CHU Bordeaux, 33000 Bordeaux, France; 4 Neurosurgery Department, CHU Bordeaux, 33000 Bordeaux, France

**Keywords:** Non-functioning pituitary adenoma, Radiotherapy, Surgery, Wait and see

## Abstract

**Introduction:**

The first line of treatment for nonfunctioning pituitary adenoma (NFPA) is surgery. Adjuvant radiotherapy or surveillance and new treatment (second surgical operation or salvage radiotherapy) in case of recurrence are options discussed at the multidisciplinary tumor board. The purpose of this study was to evaluate the therapeutic outcome for each option.

**Methods:**

The records of 256 patients followed with NFPA between 2007 and 2018 were retrospectively reviewed. Mean age at initial surgery was 55 years [18–86]. Post-operative MRI found a residual tumor in 87% of patients. Mean follow-up was 12.1 years [0.8–42.7].

**Results:**

After initial surgery, 40 patients had adjuvant radiotherapy. At 5, 10 and 15 years progression-free survival (PFS) was significantly different after surgery alone (77%, 58% and 40%) compared to surgery and adjuvant radiotherapy (84%, 78% and 78%) (HR = 0.24 [0–0.53] p < 0.0005). Overall, after first, second or third surgical operation, 69 patients had adjuvant radiotherapy and 41 salvage radiotherapy. Five-year PFS was similar for adjuvant (90%) and salvage radiotherapy (97%) (p = 0.62). After a second surgical operation, 62% and 71% of patients were irradiated after 2 and 5 years respectively. The risk of corticotropic and thyrotropic deficiency rates were 38% and 59% after second or third surgical operation and 40% and 73% after radiotherapy. Brain tumors occurred in 4 patients: 1 meningioma present at initial surgery, and after radiotherapy, 1 neurinoma which appeared at 5 years, 1 glioblastoma at 13 years and 1 meningioma at 20 years.

**Conclusion:**

Among patients treated by surgery for NFPA, a “wait-and-see” attitude should be an option since adjuvant radiotherapy is not superior to salvage radiotherapy. However, in case of recurrence or progression, the authors recommended delivery of salvage radiotherapy to avoid a second surgical operation.

**Supplementary Information:**

The online version contains supplementary material available at 10.1186/s13014-022-02133-z.

## Introduction

The incidence of pituitary adenomas (PA) keeps increasing (3.47/100,000 in 2016 in the USA) while non-functioning pituitary adenomas (NFPA) represent about one-third of all PA [1–3]. NFPA are mostly diagnosed because of chronic compressive symptoms (visual field defects or headaches) or hypopopituarism and do not present any secreting clinical manifestation. They are mostly gonadotropic or null cell adenoma, less often adenoma with ACTH, GH, PRL or TSH immunostaining [4].

The standard treatment for symptomatic NFPA’s consists in a debulking transsphenoidal surgery, associated with a 26.5% morbidity rate [5–7]. Endoscopic endonasal transsphenoidal approach is associated with toxicity such as worsening in 2.4% of visual functions, 13.7% of pituitary functions and 6.2% permanent diabetes insipidus, 0.8% cerebrospinal fluid leaks with hard reconstructions of the sella [6]. Shorter hospital stays and fewer postoperative complications are associated with higher-volume hospitals and surgeons [7].

The risk of relapse after exclusive surgery is 15.2–66% after 5 years, 41–50.5% after 10 years and 51–72% after 15 years [8–12]. Strategy after surgery is debated [13–15]. Post-operative radiotherapy has proven to be efficacious in reducing the risk of relapse: 0–6% after 5 years, 0–9% after 10 years and 7–9% after 15 years [8, 10–12, 16–18]. However, radiotherapy sometimes causes further pituitary deficiency and may induce cerebrovascular disease, secondary intracranial tumors and psycho-cognitive dysfunction [19–24]. Nowadays, high-precision radiotherapy with MRI registration and new intensity-modulated techniques reduce toxicity, notably by hippocampal-sparing [13, 25, 26].

Clinicopathological prognostic classification that combines tumor size, type, and grade accounting for invasion and proliferation criteria could be tools to decide between surveillance and adjuvant radiotherapy [27]. In case of recurrence, a second surgical operation or salvage radiotherapy is discussed at the multidisciplinary tumor board level [14].

Our purpose was to describe treatments for NFPA following initial surgery, including further surgeries and radiotherapy, and to evaluate outcomes after these second-line treatments. Secondary toxicities, especially endocrinological, as well as deciding factors that lead to a decision for radiotherapy were also studied.

## Materials and methods

All patients followed (day hospital endocrinology department, radiotherapy department, neurosurgery department) for clinical NFPA at the Bordeaux University Hospital between January 2007 and January 2018 were screened. The cut-off date for the data was fixed at January 1st, 2021. Inclusion criteria were: age ≥ 18 years old, first-line surgery, histologically proven benign pituitary adenoma, > 6 months follow-up. Exclusion criteria were: pituitary carcinoma, NFPA without initial surgery or cerebral radiotherapy before surgery. Progression was defined as the need for further treatment (radiotherapy or surgery). Each treatment modification was decided by the multidisciplinary tumor board including neurosurgeon, radiation oncologist, neuroradiologist and endocrinologist. Salvage treatment was decided in case of objective radiological progression on MRI. Surgery was preferred in case of visual disorder. Salvage radiotherapy was preferred for patients with cavernous invasion and/or without new optic deficiency. Adjuvant radiotherapy consisted of irradiation within 6 months after surgery indicated by residual tumor or histoprognostical considerations. Salvage radiotherapy was defined as irradiation indicated by tumor evolution during follow-up, after surgery. The decision for adjuvant radiotherapy was taken by the multidisciplinary tumor board. Factors associated with adjuvant decision making will be studied. Adjuvant or salvage radiotherapy was done with standard fractionation, at 1.8 Gy to 2 Gy/fraction. In our center, the usual prescribed dose is 48.6 Gy at 1.8 Gy/fraction, 5 days per week, during 5 weeks and 2 days. To maintain a homogeneous treatment schedule, radiosurgery and hypofractionation radiotherapy, for instance with 5 fractions, were excluded. All data were collected retrospectively and in compliance with institutional ethical policies. Based on the documents presented, the publication group of the Ethics Committee of the Bordeaux University Hospital issued a favorable opinion to publish this research (CE-GP-2019/19).

### Tumor characteristics

The vast majority of resected tumors were analyzed in the same laboratory. All immuno-histochemical analysis results (ACTH, GH, PRL, TSH, FSH, and sub-unit α2, β-FSH, β-LH) were available except for patients operated before 2000.

Null cell adenomas were defined as tumors without any pituitary hormones or other immunomarkers [28]. NFPAs were classified according to the HYPOPRONOS clinicopathological classification of pituitary endocrine tumors [27].

### Follow-up and statistical analysis

As recommended, the postoperative residual tumor status was analyzed following a 3–12 months post-operative MRI [29]. Prior to any treatment decision, all patients were assessed by a multidisciplinary tumor board including endocrinologists, neurosurgeons, neuroradiologists, and radiation oncologists.

All statistical analyses were carried out using XLSTAT® software. Chi-square analysis was used to compare categorical variables, t-test to compare continuous variables, and Mann–Whitney test to compare continuous variables concerning time ranking. Two-sided p-values below 0.05 were considered statistically significant. Survival analysis was done according to the Kaplan Meier method and compared according to the Cox model.

## Results

The mean follow-up after initial surgery was 12.1 years [0.8–42.7]. After 3, 5, 10, 15 and 20 years, 243 (95%), 210 (82%), 127 (49%), 68 (27%) and 24 (9%) patients were followed. The first patient initially underwent surgery in February 1978. Most patients (n = 204; 79.6%) were treated after year 2000.

### Patients and tumor characteristics

Two hundred and fifty-six patients met the inclusion criteria (Table 1).

**Table 1:** Patient and tumor characteristics

Patients	Total
n	256
Mean age at diagnoses	55 (18–86)
Sex ratio (M/F)	1.42 (150/106)
Mean follow up after surgery (years)	12.1 (0.8–42.7)
*Pre-operative data*	
Symptoms at diagnoses	
Visual disorders	157/234 (67%)
Headache	74/233 (32%)
Fortuitous	43/236 (18%)
Adenoma size	
Maximal tumor height (mm)	28.6 (12–60)
Microadenoma (< 1 cm)	0
Macroadenoma (1–4 cm)	133/148 (90%)
Giant adenoma (> 4 cm)	15/148 (10%)
Patients with cavernous sinus invasion	86/123 (70%)
Post-operative data	
Transsphenoidal/Transcranial surgery	142/11
Residual tumor	199/228 (87%)
Mean maximal tumor height (mm)	12.4 (0–50)
Apoplexy	24/230 (10%)
Postoperative deficiencies	
Corticotropic	73/218 (35%)
Thyrotropic	108/220 (49%)
Gonadotropic	99/217 (46%)
Panhypopuitarism	39/221 (18%)
Diabetes insipidus	12/218 (6%)
Normalization of preoperative visual disorders	
Unknown	47 (18.4%)
Complete	86 (33.6%)
Partial	42 (16.4%)
No improvement	16 (6%)
None initial	65 (25.4%)
Patients with cavernous sinus invasion	82/195 (42%)
Tumor type	
Unknown	26 (10.2%)
Nude cells	93 (36.3%)
ACTH	13 (5%)
FSH/LH	106 (41.4%)
GH	0 (0%)
PRL	4 (1.6%)
TSH	1 (0.4%)
Pluri-hormonal dominance	13 (5.1%)
*Grades*	
Unknown	*128/256*
1a	26/128 (20%)
1b	24/128 (19%)
2a	40/128 (31%)
2b	38/128 (30%)

In case of data unavailable, the total of evaluable patients is indicated. For instance, preoperative visual disorder was evaluable in 234 patients

Mean age at diagnosis was 55 years, and there were 106 women for 150 men. Visual disorder (67%) and headache (32%) were the most frequent symptoms at diagnosis.

NFPAs were macroadenomas (between 10 and 40 mm) (90%) and giant adenomas (over 40 mm) (10%), with a 28.6 mm [12–60] mean size. Before the first surgery, 70% of patients (86/123 evaluable) presented at least one cavernous sinus invasion and 25% both. After surgery, 87% of patients presented an MRI-residual tumor. Invasion of at least one cavernous sinus concerned 42% of patients (82/195 evaluable).

NFPA were gonadotroph (n = 106, 41.4%), null-cell (n = 93, 36.3%), corticotroph (n = 13, 5%), with pluri-hormonal dominance (n = 13, 5.1%), lactotroph (n = 4, 1.6%), thyrotroph (n = 1, 0.4%) unknown type (n = 26, 10.2%) and no somatotroph adenoma. According to the HYPOPRONOS clinico-pathological classification of pituitary endocrine tumors, on available data, tumors were classified for 20% as grade 1a, 19% grade 1b, 31% grade 2a and 30% grade 2b.

### Treatment sequence

**Fig. 1:**
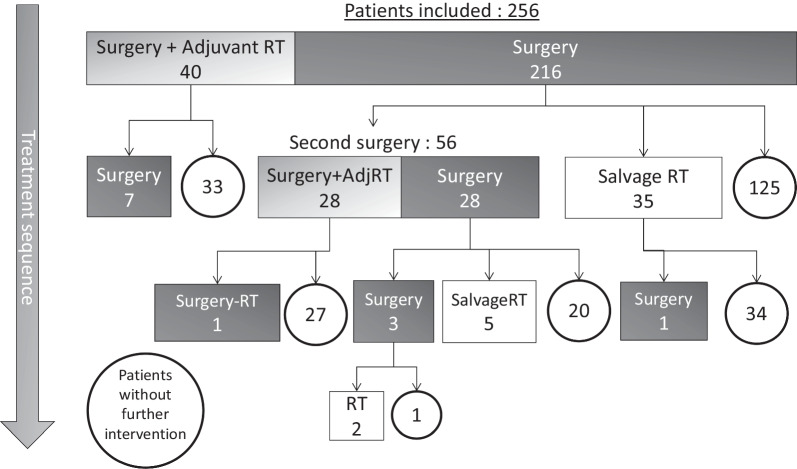
Flow chart of treatment sequence for the entire cohort of patients. *S* surgery, *aRT* adjuvant radiotherapy, *sRT* salvage radiotherapy. Patients without further intervention are indicated in the circles

Among 256 patients treated by surgery, 40 patients underwent adjuvant radiotherapy and 216 patients underwent wait-and-see attitude. 125 patients (49%) were free of further treatment during the entire follow-up whereas 91 patients underwent a salvage intervention: 35 patients underwent salvage radiotherapy, 56 patients underwent a second surgical operation of whom 28 patients with adjuvant radiotherapy (Fig. 1).

One hundred and ten (43%) actually received radiotherapy. Adjuvant treatment was indicated for 69 patients: 40 following initial surgery and 28 following a second surgical operation and 1 after a third surgical operation. Salvage treatment was indicated in 41 patients: 35 following initial surgery, 5 following a second surgical operation, and 1 after a third surgical operation. After radiotherapy, 9 patients out of 110 underwent another surgical operation.

After a second surgical operation without adjuvant radiotherapy, 5 patients out of 28 underwent salvage radiotherapy, and 3 underwent a third surgical operation.

No patient received any medical treatment such as dopamine agonist, somatostatin analog nor temozolomide.

### Adjuvant radiotherapy after first surgery

Among the 40 patients who underwent surgery and adjuvant radiotherapy, 100% had a surgical remnant compared to 85% among 216 who did not underwent adjuvant radiotherapy (p = 0.02) (Additional file 2: Table S1). In the surgery with adjuvant radiotherapy group, patients were also younger (49 years vs. 56 years, p = 0.006) than in the other group, there was more cavernous sinus invasion (65% vs. 39%, p = 0.02), adenoma size before surgery was bigger (33 mm vs. 28 mm, p = 0.025). At last, median treatment period for adjuvant radiotherapy was November 2000 compared to October 2009 for the other group (p < 0.001). In our serie, adjuvant radiotherapy was more indicated in the past compared to nowadays.

### Treatment in wait and see sequence after first surgery

Factors associated with failure and salvage treatments were studied for patients without adjuvant radiotherapy (Table 2).

**Table 2:** Factors associated with salvage treatments after first surgery without adjuvant radiotherapy

	Surgery alone (S)	Surgery–surgery (S–S)	Surgery–sRT (S–sRT)	
Total (% out of 216)	125 (58%)	56 (26%)	35 (16%)	
Gender				
Female (%)	56 (45%)	23 (41%)	16 (46%)	p = 0.87
Male (%)	69 (55%)	33 (59%)	19 (54%)	
Mean age at first surgery				
Years	57.2	50.1	59.3	p = 2.10^−3^ (S vs. S–S)
				p = 0.4 (S vs. S–sRT)
				p = 10^−3^ (S–S vs. S–sRT)
Visual disorders				
No	48 (41%)	16 (32%)	9 (27%)	p = 0.27 (S vs. S–S)
Yes	69 (59%)	34 (68%)	24 (73%)	p = 0.15 (S vs. S–sRT)
Unknown	8	6	2	p = 0.64 (S–S vs. S–sRT)
Cavernous sinus invasion				
No	87 (74%)	12 (39%)	6 (25%)	p < 10^−3^ (S vs. S–S)
Yes	30 (26%)	19 (61%)	18 (75%)	p < 10^−5^ (S vs. S–sRT)
Unknown	8	25	11	p = 0.28 (S–S vs. S–sRT)
Tumor type				
Nude cells	40 (37%)	20 (45%)	14 (44%)	p = NS
ACTH	5 (5%)	3 (7%)	1 (3%)	
FSH/LH	62 (57%)	21 (48%)	17 (53%)	
GH	0	0	0	
PRL	1 (1%)	0	0	
TSH	1 (1%)	0	0	
Residual tumor after first surgery				
No	26 (22%)	3 (6%)	0 (0%)	p < 0.05 (S vs. S–S)
Yes	92 (78%)	45 (94%)	31 (100%)	p = 4.10^−3^ (S vs. S–sRT)
Unknown	7	8	4	p = 0.16 (S–S vs. S–sRT)
Mean duration between first surgery and salvage treatment				
Years	NA	6.6	5.4	p = 0.23

*S* surgery, *sRT* salvage radiotherapy, *NA* not applicable

Two factors were significantly associated with second surgery and salvage radiotherapy, compared to first surgery alone: cavernous sinus invasion and residual tumor after surgery. Otherwise, patients with 2 surgeries were significantly younger (mean age: 50.1 years) than patients treated by only one surgery (mean age: 57.2 years) and patients needing salvage radiotherapy (mean age: 59.3 years). The mean time between the first and second surgery was 6.3 years.

### Progression-free survival

**Fig. 2:**
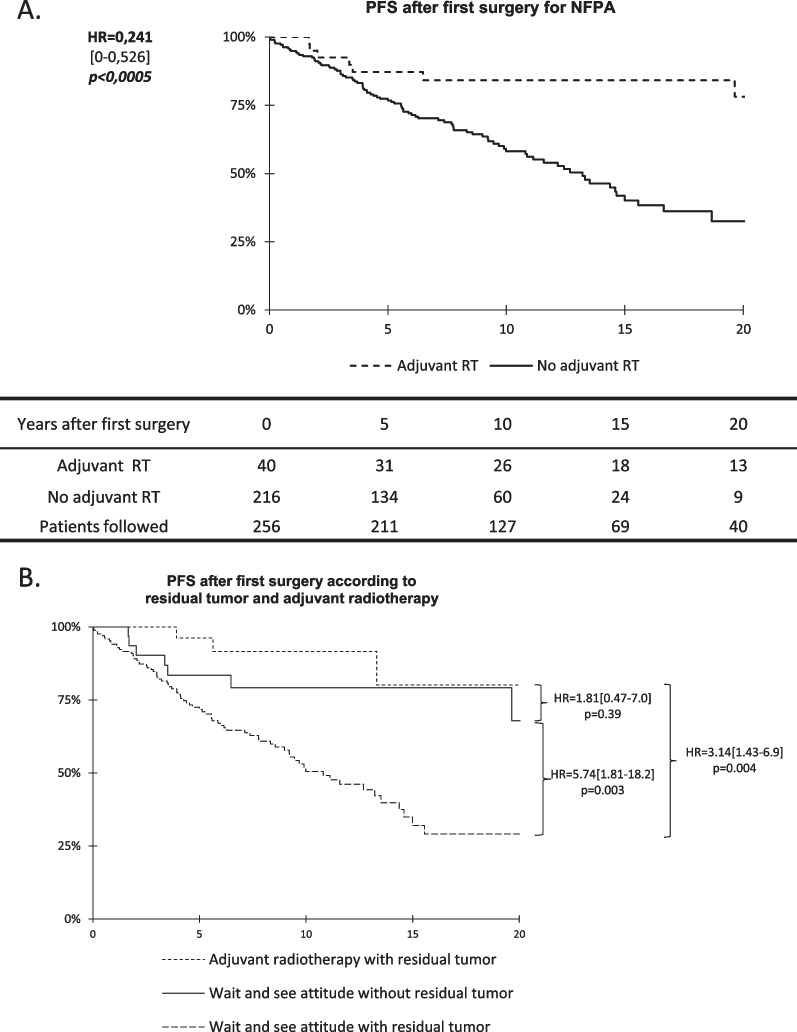
**A** PFS after initial surgery for the whole cohort. Patients exposed to relapse are shown in lines 1 and 2, and patients followed are shown in third line. **B** PFS for patients with available data for post-operative residual tumor after first surgery (N = 228). Patients could have wait and see attitude and had post-operative residual tumor (N = 168) or without residual tumor (N = 29) or could be treated with adjuvant RT, all with residual tumor (N = 31). No patient treated by adjuvant radiotherapy had residual tumor. Patients without information concerning their residual tumor were excluded from this analysis (N = 28)

Five-year, 10-year and 15-year PFS was respectively 77%, 58% and 40% for patients treated by surgery alone compared to 84% and 78% after 5, 10 and 15 years (HR = 0.24 [IC95% 0–0.53]; p < 0.0005) for patients treated by surgery followed by adjuvant radiotherapy (Fig. 2A). Explorative analysis showed that patients with residual tumor and adjuvant radiotherapy had better PFS than unirradiated patients (HR = 3.14 [1.43–6.9] p = 0.004) (Fig. 2B). Moreover unirradiated patients had a worse PFS if they had a residual tumor (HR = 5.74 [1.81–18.2] p = 0.003). PFS was not different between patients with residual tumor and adjuvant radiotherapy, and patients without residual tumor unirradiated. At 10 years, PFS for patients treated by adjuvant radiation and for unirradiated patients without and with residue was respectively 91.6%, 79.2% and 50.5%.

**Fig. 3:**
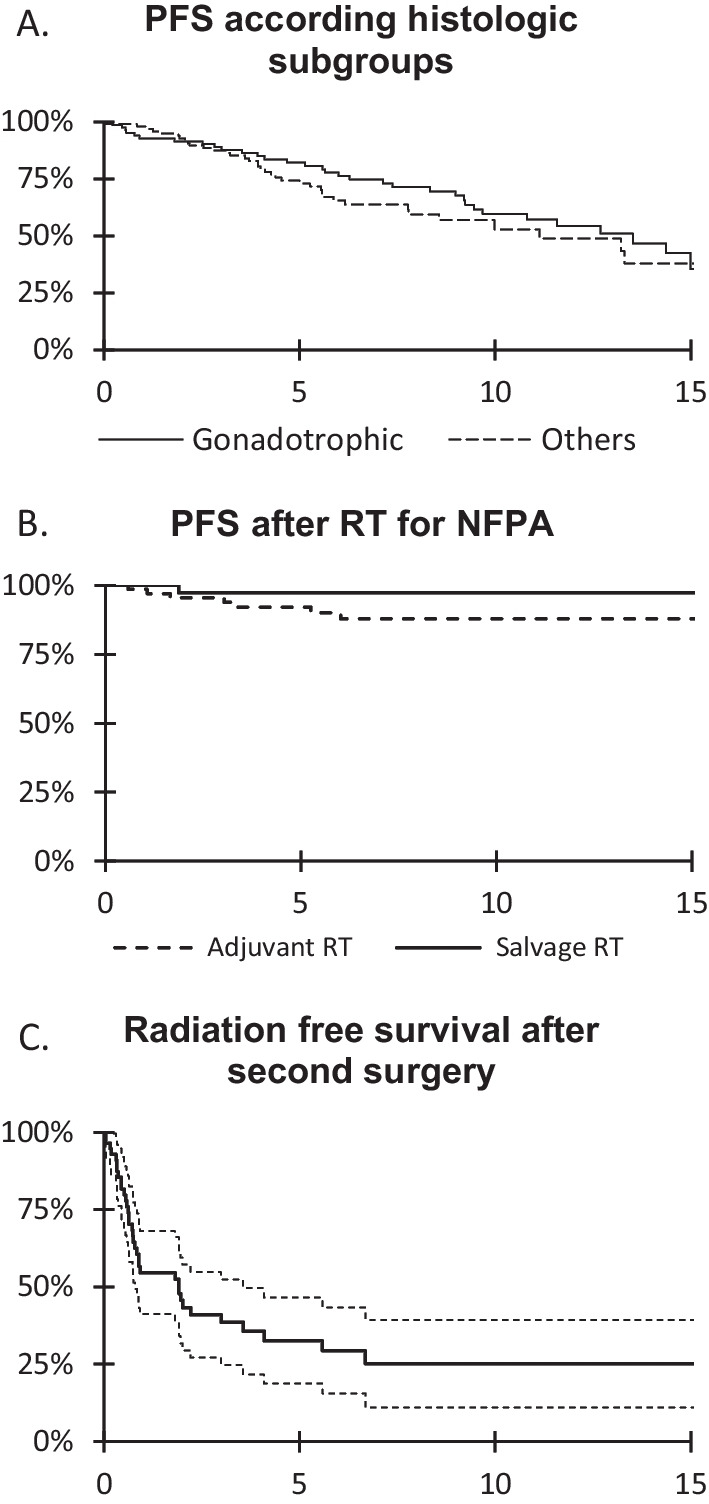
**A** PFS according to histological subtypes: Gonodotrophic: 99 vs. other subtypes (null-cell = 74, corticotroph = 9, plurihormonal = 12, lactotroph = 1, thryreotroph = 1) (p = 0.285) **B** PFS after adjuvant RT or salvage RT is no different (p = 0.17) **C** Radiation-free survival after second surgical operation

Among 196 patients with available immunohistochemistry and treated by surgery without adjuvant irradiation, PFS for gonadotrophic NFPA (N = 99) was no different compared to other subgroups (null-cell = 74, corticotroph = 9, plurihormonal = 12, lactotroph = 1, thryreotroph = 1)(p = 0.28) (Fig. 3A). Five-year PFS was 90% in patients treated by adjuvant radiotherapy compared to 97% in patients treated by salvage radiotherapy for recurrence (p = 0.62) (Fig. 3B). Radiotherapy free survival after a second surgical operation was 38% after 2 years, 29% after 5 years and 26% after 10 years (shown in Fig. 3C).

Radiotherapy was significantly more prescribed in case of a rapid recurrence estimated by the time between two surgical operations (p = 0015, when considered as a continuous variable in Cox model), particularly with a 2-year cutoff (HR = 0.31 [IC95% 0–0.62] p = 0.001) (shown in Additional file 1: Fig. S1).

### Radiotherapy

Among 110 irradiated patients, radiotherapy dose and fractionation data were available for 94 patients (85%). Radiotherapy was delivered according to standard daily fractionation, five days a week. The most common dose prescribed was 48.6 Gy (27 × 1.8 Gy) in 80 patients (85%). Other schemes were 45–51 Gy with 1.8–2.2 Gy per fraction.

Among 107 patients irradiated, 91 (83%) were treated in one center. The radiotherapy modalities evolved over time: except for one patient (1%) treated with a ^60^Co source in 1989, all 25 patients (27%) treated before 2007 received conformational non stereotactic radiotherapy. All other patients (71%) received “modern” radiotherapy technique as defined by Minniti et al. [13]. Since 2008, 24 patients (26%) had conformational stereotactic treatment. Since 2012, 20 patients (22%) were treated by intensity-modulated stereotactic radiotherapy and then 21 patients (23%) with non-coplanar arcs allowing for hippocampal sparing [26].

### Hormonal deficiencies

Postoperative deficiencies concerned corticotropic, thyrotropic and gonadotrophic axis for respectively 35%, 49% and 46% of patients. GH deficiencies as well as pre-operative deficiencies were not systematically recorded. Among the patients who were treated with only one surgery, 42%, 52% and 48% of patients had corticotropic, thyrotropic and/or gonadotropic deficiencies. Irradiated patients had corticotropic, thyrotropic and/or gonadotropic deficiencies in respectively 40%, 73% and 63% of cases. Forty-one percent of patients (42/103) suffered from panhypopituitarism after surgery and radiotherapy with a 14.6-year mean follow-up (1.1–42.7). After two to three surgical operations, post-operative deficiencies on corticotropic, thyrotropic and gonadotrophic axis accounted for respectively 38%, 59% and 60% of patients (Additional file 3: Table S2).

### Non-hormonal toxicities

No radiation-induced optic neuropathy, nor any nerve toxicity were reported. Similarly no grade > 2 acute toxicities were reported following radiotherapy whereas 9 grade 2 toxicities (7 asthenia and 2 headaches) were reported. One patient had a meningioma diagnosed at initial surgery, and 3 patients had cerebral tumors after radiotherapy: a neurinoma (after 2 years), a glioblastoma (after 13 years) and a meningioma (after 20 years).

Among 256 patients, 19 patients (7.5%) had a stroke without any significant difference between irradiated patients (9%) and non-irradiated patients (6%) (p = 0.47, NS).

## Discussion

### After initial surgery: “wait-and-see” attitude

We present here one of the largest postoperative NFPA studies and the first to specify the timeline of postoperative management. Among 256 surgically treated NFPA patients, 60% needed a second treatment in a 15-year follow-up.

A complete NFPA surgery is difficult because of its anatomic position: resections are often sub-complete. The pooled results of 12 studies including 1455 patients suggest a 57% MRI residual tumor rate after surgery reaching 70% in the most recent study whereas we even found an 87% (199/228) post-operative residual tumor rate [12, 23]. These high rates could justify the indication for adjuvant radiotherapy. After exclusive surgery, Chen et al. reported a PFS of 71% at 5 years and 59% at 10 years, compared with our own 77%, 58% at 5 and 10 years [30].

Conversely, the combination of surgery and radiotherapy shows good and consistent efficacy with relapse rates between 0 and 6% after 5 years, 0 and 9% after 10 years and 6 and 9% after 15 years [8, 10–12, 18]. In our series, the relapse rate was 10% at 5 years for patients after adjuvant radiotherapy. Adjuvant radiotherapy might have been a good option after surgery.

However, radiotherapy could be responsible for late toxicities such as pituitary deficiencies that remain important sequelae after surgery and radiotherapy. Our results showing 32% and 48% of corticotropic and thyrotropic deficiencies after surgery and 46% and 58% after surgery and radiotherapy are consistent with the existing literature [19, 23]. The self-reported quality of life is stable or improved in almost all patients [31].

In addition, radiotherapy is thought to increase the risk of long-term cerebrovascular accidents by a 2–4.1 factor according to most studies [32, 33]. Another study denies this risk, based on a review of 11 studies and 4394 patients [22]. Our study did not observe a significantly increased stroke risk estimated at 6–9%. Radiotherapy is also believed to cause second tumors as redefined by Sheehan et al. [34]. We found 3 brain tumors in our cohort, including 2 out of 110 after radiotherapy despite a 12-year mean postoperative follow-up. Previously, Sheehan et al. described no second tumor among 1621 patients while Brada et al. with five patients, mention a 1.3% and 1.9% 10- and 20-year cumulative risk [24, 34]. Burman et al. based on 8917 adenoma analyses, including 3751 NFPA and 3236 radiotherapy treatments, found a 3.3-fold higher relative risk of developing de novo malignant brain tumors and a fourfold higher risk of meningiomas in patients who received radiotherapy [35]. Our results corroborate the idea that, if developing a brain tumor after radiotherapy is possible, the risk remains nevertheless low and delayed.

Most NFPA has a slow growth rate with a 3.4-year mean doubling time, without radiotherapy [30]. Nowadays, it appears that advances in pituitary imaging and generalization of multidisciplinary tumor boards are also tools to explore tumor progression precisely to avoid emergency surgery.

It is admitted that deferring radiotherapy is a safe attitude in case of complete resection if the patient is included in an MRI surveillance protocol [10, 14]. Therefore, a radiological relapse will rarely need immediate treatment [36].

Differing radiotherapy with a “wait-and-see” attitude appears all the more reason for all patients and particularly in non-deficient patients and women of childbearing age (gonadic deficiency, expensive and imperfect lifetime substitution treatment). Conversely, for patients already suffering from hormonal deficiencies, differed radiotherapy would induce no hormonal preservation benefits.

Ferrante et *al.* studied as we did the time leading up to new medical treatment [37]. Their cohort included 98% of patients treated by surgery and 41% who had adjuvant radiotherapy. With a mean follow-up of 9.3 years, 73 relapses out of 226 patients (32%) occurred. In the group of patients treated by surgery alone, the mean time before the second surgical operation was 5.2 years (± 4.7 years) consistent with our results (6.3 years). This time interval was compatible with a “wait-and-see” attitude. Even in the population of patients with residual tumor, we found in our serie that more than half of patients (50.5%) will not need further treatment (surgery or salvage radiotherapy) at 10 years.

In a multicenter matched cohort study, Pomeraniec et al. compared early (< 6 months) and late (> 6 months after resection) radiation by Gamma Knife after NFPA resection [38]. At 4 years, radiological progression was 6.1% in the early radiation group vs. 14.3% in the late radiation group. In this study, new endocrinopathies were constated in 27–30% of people during the follow-up period. In our study, after the first surgery, 77% and 58% of patients were free from new treatment (surgery/radiotherapy) at 5 and 10 years respectively. Increasing the radiological efficacy by 8.2% at 4 years does not seem to us sufficient to favor an adjuvant treatment which will cause 1 patient out of 3 or 4 to have permanent endocrine treatment.

### Salvage radiotherapy in case of progression

Interestingly, fractionated radiotherapy was as effective when used as adjuvant or as salvage treatment, which is consistent with other published results [10, 13, 39]. Pomeraniec et al. showed less radiological progression after early radiation, but it is difficult to know if there is a clinical impact in the available data [38]. Regarding radiotherapy, in our series, radiation technique is consistent with most treatments at a dose of 48.6 Gy [45–50.4] at 1.8–2 Gy/fraction. This dose conformed with Grigsby et *al.* who showed a dose–response relationship with a better local control above 45 Gy over a three-decade dose-escalation experience after prior surgery [40]. This could also be a limitation because radiosurgery is preferred by many for low treatment volumes not too close to the optic tract. However, radiosurgery has a cost in cranial nerve deficit risk (0.8–6.6%) with mixed reports in the existing literature [41–43]. For instance, Cifarelli et al. described 4% of dysfunction in 217 pituitary adenomas treated by Gammaknife, one-third of which was permanent [44]. Regarding optic nerve toxicity, a review of historical series of fractioned radiotherapy found an incidence of 11 radiation optic neuropathies out of 2063 treated patients (0.53%) [45]. More recently, no radiation-induced neuropathy has been reported in 76 patients treated with fractionated radiotherapy (50.4 Gy/1.8 Gy/fraction) for pituitary adenoma with a minimum follow-up of 5 years [46]. Standard fractionation is not limited by size or optic nerve proximity, and our results seem to validate this approach. Efficacy of fractionated radiotherapy and stereotactic is judged to be not different [13, 15]. As a matter of fact, a comparison of fractionated radiotherapy and radiosurgery suggests that radiosurgery should be preferred for small tumors away from the optic chiasma, whereas fractioned stereotactic is more convenient for > 2.5–3 cm tumor or adenomas located near optic pathways [47].

Stereotactic radiotherapy in 3–5 fractions could be proposed for tumors located near visual pathways if radiosurgery is contraindicated. This possibility needs to be demonstrated by long term safety and efficacy studies [13].

Only randomized trials could recommend one or another technique in this pathology with a low relapse rate. In conclusion, salvage radiotherapy gives excellent results with 97% PFS at 5 years, and should be preferred to adjuvant radiotherapy.

After a second surgical operation, in our series, 62% of patients had radiotherapy within 2 years, and 71% within 5 years. This surgical intervention is often required to decompress the optic tract but it is not a way to avoid radiotherapy.

### Study limitation

Our study presents some intrinsic limitations. Even if this study has one of the longest follow-up in the literature, it is monocentric and retrospective. Stereotactic radiosurgery was not available until recently and all patients were treated with conventional fractionation, even if most then were treated by “modern” radiotherapy. Only functioning adenomas were addressed to radiosurgery by GammaKnife®. The main limitation of this study is that we did not have access to the indication of radiotherapy or further surgeries. Finally, only partial data from endocrinologic status were available limiting interpretation of surgery and radiotherapy impact on it.

## Conclusion

Over a long follow-up of 256 patients with operated NFPA, we reported that 60% of patients needed further treatment in a 15-year follow-up time. Better PFS above 90% at 5 years was similarly reported using adjuvant or salvage radiotherapy. Follow-up with MRI justifying a “wait-and-see” attitude. We consider that a watch and wait attitude after initial surgery is a good option. Even in a population of patients with residual tumor, more than half of patients will not need further treatment at 10 years. This is compatible with a “wait-and-see” attitude. If this second treatment consisted of surgery, about 3/4th of patients would receive radiotherapy within the next 5 years, whereas after radiotherapy less than 10% needed surgery within the next 5 years. We recommend that when significant growth is observed, leading to a risk of surgery within the following years, salvage radiotherapy should be proposed to reduce progression but carries with it some risks that must be weighed for individual patients alongside other variables like age, endocrine function, and residual tumor size/location to make personalized decisions for individual patients.

## Supplementary Information

**Additional file 1. Fig. S1**: Radiation free survival according to duration between 2 surgical operations. It is statistically significant when this duration is considered as a continuous variable (p=0.015) or with a cut-off at 2 years (p=0.001).

**Additional file 2. Table S1**: Patient and tumor characteristics according to treatment strategy.

**Additional file 3. Table S2**: Final deficiencies according to intervention. (Radiotherapy encompasses adjuvant and salvage radiotherapy).

## Data Availability

The datasets used and/or analyzed during the current study are available from the corresponding author on reasonable request.
