# Supplementary material for: Management after initial surgery of nonfunctioning pituitary adenoma: surveillance, radiotherapy or surgery?
Source: Radiat Oncol. 2022 Oct 13;17:165. doi: 10.1186/s13014-022-02133-z (PMC9559766; doi:10.1186/s13014-022-02133-z)
Supplement: Supplementary file 1 — Additional file 1. Fig. S1: Radiation free survival according to duration between 2 surgical operations. It is statistically significant when this duration is considered as a continuous variable (p=0.015) or with a cut-off at 2 years (p=0.001). [file 13014_2022_2133_MOESM1_ESM.pptx]

## Slide 1
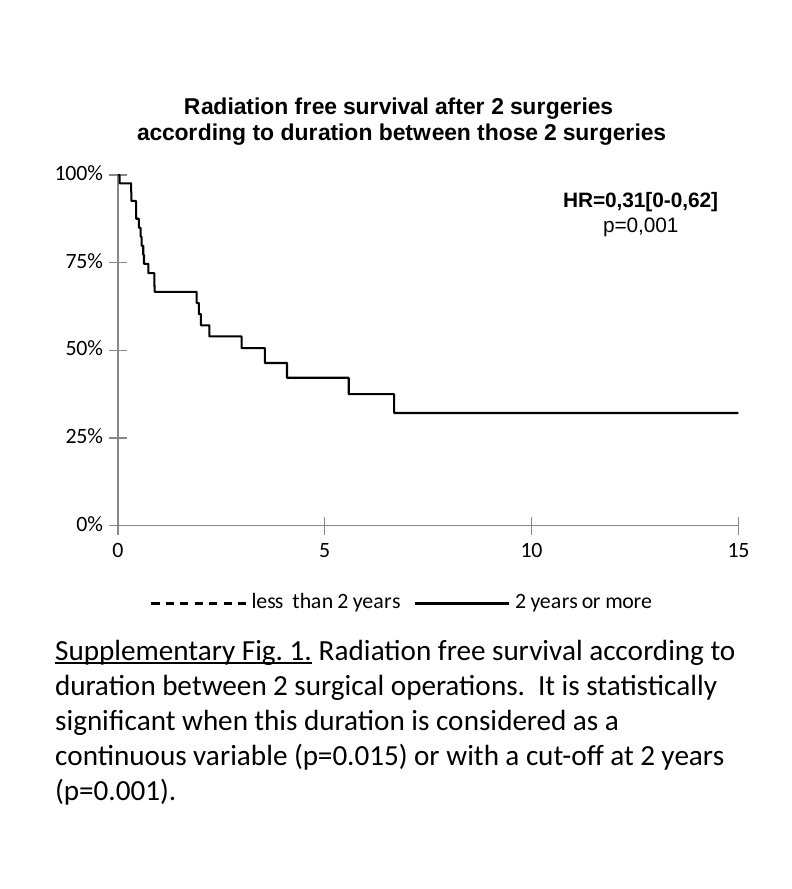

### Chart: Radiation free survival after 2 surgeries
according to duration between those 2 surgeries
| Category | less than 2 years | 2 years or more |
|---|---|---|HR=0,31[0-0,62]
p=0,001
Supplementary Fig. 1. Radiation free survival according to duration between 2 surgical operations. It is statistically significant when this duration is considered as a continuous variable (p=0.015) or with a cut-off at 2 years (p=0.001).
