# Supplementary material for: Management after initial surgery of nonfunctioning pituitary adenoma: surveillance, radiotherapy or surgery?
Source: Radiat Oncol. 2022 Oct 13;17:165. doi: 10.1186/s13014-022-02133-z (PMC9559766; doi:10.1186/s13014-022-02133-z)
Supplement: Supplementary file 2 — Additional file 2. Table S1: Patient and tumor characteristics according to treatment strategy. [file 13014_2022_2133_MOESM2_ESM.doc]

|  | **Surgery** | **Surgery - Adjuvant Radiotherapy** | **Total** |  |
| --- | --- | --- | --- | --- |
| **N** | 216 | 40 | 256 |  |
| **Mean age at diagnosis** | 56 (18 - 86) | 49 (18 - 53) | 55 (18 - 86) | p=0.006* |
| **Sex ratio (M/F)** | 121/95 | 29/11 | 1,42 (150/106) | p=0.052 |
| **Median surgery date (month/year)** | 10/2009  (08/1980 - 12/2016) | 11/2000  (02/1978 - 01/2018) | 05/2009  (02/1978 - 01/2018) | **p<0.001 **** |
| **Mean follow up after surgery (years)** | 11,1 (0,8 - 39,9) | 17,3 (3 – 42,7) | 12,1 (0,8 - 42,7) | **p<0.001 **** |
| **Pre-operative data** | | | | |
| **Symptoms at diagnoses** |  |  |  |  |
| **Visual disorders** | 127/200 (64%) | 30/34 (88%) | 157/234 (67%) | **p=0.004** |
| **Headache** | 64/199 (32%) | 10/34 (29%) | 74/233 (32%) | p=0.75 |
| **Fortuitous** | 40/202 (20%) | 3/31 (10%) | 43/233 (18%) | p=0.17 |
| **Adenoma size** |  |  |  |  |
| **Mean maximal tumor height (mm)** | 28 (12 - 60) | 33,3 (22 – 50) | 28,6 (12 - 60) | **p=0.025*** |
| **Macroadenoma (1 - 4 cm)** | 117/130 (90%) | 16/18 (89%) | 133/148 (90%) | p=0.88 |
| **Giant adenoma (> 4 cm)** | 13/130 (10%) | 2/18 (11%) | 15/148 (10%) | p=0.88 |
| **Patients with cavernous sinus invasion before surgery** | 76/109 (70%) | 10/14 (71%) | 86/123 (70%) | p=0.90 |
| **Post-operative data** | | | | |
| **Transsphenoidal / Transcranial surgery** | 127/9 | 15/2 | 142/11 | p=0.43 |
| **Residual tumor** | 168/197 (85%) | 31/31 (100%) | 199/228 (87%) | **p=0.02** |
| **Mean maximal tumor height (mm)** | 11 (0 - 50) | 25 (3 - 36) | 12,4 (0 - 50) |  |
| **Apoplexy** | 23/202 (11%) | 1/27 (4%) | 24/230 (10%) | p=0.22 |
| **Normalization of postoperative visual disorders** | | | | |
| **Complete** | 81/184 (44%) | 5/25 (20%) | 86/209 (41%) | **p=0.02** |
| **Partial** | 27/184 (15%) | 15/25 (60%) | 42/209 (20%) | **p<0.0001** |
| **No improvement** | 14/184 (7%) | 2/25 (8%) | 16/209 (8%) | p=0.94 |
| **None initial** | 62/184 (34%) | 3/25 (12%) | 65/209 (31%) | **p=0.01** |
| **Patients with cavernous sinus invasion post surgery** | 67/172 (39%) | 15/23 (65%) | 82/195 (42%) | **p=0.02** |
| **Tumor type** | | | | |
| **Nude cells** | 74/196 (38%) | 19/34 (56%) | 93/230 (40%) | **p=0.047** |
| **ACTH** | 9/196 (5%) | 4/34 (12%) | 13/230 (6%) | p=0.09 |
| **FSH/LH** | 99/196 (51%) | 7/34 (21%) | 106/230 (46%) | **p=0.001** |
| **GH** | 0 | 0 | 0/230 (0%) |  |
| **PRL** | 1/196 (1%) | 3/34 (9%) | 4/230 (2%) | **p<0.001** |
| **TSH** | 1/196 (1%) | 0 | 1/230 (0.4%) | p=0.68 |
| **Pluri-hormonal dominance** | 12/196 (6%) | 1/34 (3%) | 13/230 (6%) | p=0.46 |
| **Grades** | | | | |
| **1a** | 24/111 (22%) | 2/17 (12%) | 26/128 (20%) | p=0.35 |
| **1b** | 14/111 (13%) | 10/17 (59%) | 24/128 (19%) | **p<0.0001** |
| **2a** | 38/111 (34%) | 2/17 (12%) | 40/128 (31%) | p=0.06 |
| **2b** | 35/111 (32%) | 3/17 (18%) | 38/128 (30%) | p=0.24 |

**Supplementary Table 1.** Patient and tumor characteristics according to treatment strategy.

*Khi2 test was used for all categorical variables *indicates t-test ** indicates Mann Whitney test.*
