# Supplementary material for: Management after initial surgery of nonfunctioning pituitary adenoma: surveillance, radiotherapy or surgery?
Source: Radiat Oncol. 2022 Oct 13;17:165. doi: 10.1186/s13014-022-02133-z (PMC9559766; doi:10.1186/s13014-022-02133-z)
Supplement: Supplementary file 3 — Additional file 3. Table S2: Final deficiencies according to intervention. (Radiotherapy encompasses adjuvant and salvage radiotherapy). [file 13014_2022_2133_MOESM3_ESM.doc]

**Supplementary Table 2.** Final deficiencies according to intervention. (Radiotherapy encompasses adjuvant and salvage radiotherapy)

| **Hormonal deficit** | **One surgery** | **Surgery (1 to 3) and radiotherapy** | **Surgeries without radiotherapy** | **Total** |
| --- | --- | --- | --- | --- |
| **Total** | 125 | 110 | 21 | 256 |
| **Corticotropic** | 37/125 (42%) | 41/102 (40%) | 6/16 (38%) | 84/243 (35%) |
| **Thyrotropic** | 65/125 (52%) | 75/103 (73%) | 10/17 (59%) | 150/245 (61%) |
| **Gonadotropic** | 60/124 (48%) | 63/100 (63%) | 9/15 (60%) | 132/239 (51%) |
